# Motor Cortex Neuroplasticity Following Brachial Plexus Transfer

**DOI:** 10.3389/fnhum.2013.00500

**Published:** 2013-08-19

**Authors:** Stefan Dimou, Michael Biggs, Michael Tonkin, Ian B. Hickie, Jim Lagopoulos

**Affiliations:** ^1^Sydney Medical School, The University of Sydney, Sydney, NSW, Australia; ^2^North Shore Private Hospital, St Leonards, NSW, Australia; ^3^Brain and Mind Research Institute, The University of Sydney, Sydney, NSW, Australia

**Keywords:** brachial plexus transfer, phantom limb, motor cortex, neuroimaging, MRI

## Abstract

In the past decade, research has demonstrated that cortical plasticity, once thought only to exist in the early stages of life, does indeed continue on into adulthood. Brain plasticity is now acknowledged as a core principle of brain function and describes the ability of the central nervous system to adapt and modify its structural organization and function as an adaptive response to functional demand. In this clinical case study we describe how we used neuroimaging techniques to observe the functional topographical expansion of a patch of cortex along the sensorimotor cortex of a 27-year-old woman following brachial plexus transfer surgery to re-innervate her left arm. We found bilateral activations present in the thalamus, caudate, insula as well as across the sensorimotor cortex during an elbow flex motor task. In contrast we found less activity in the sensorimotor cortex for a finger tap motor task in addition to activations lateralized to the left inferior frontal gyrus and thalamus and bilaterally for the insula. From a pain perspective the patient who had experienced extensive phantom limb pain (PLP) before surgery found these sensations were markedly reduced following transfer of the right brachial plexus to the intact left arm. Within the context of this clinical case the results suggest that functional improvements in limb mobility are associated with increased activation in the sensorimotor cortex as well as reduced PLP.

## Introduction

The case involves a 27-year-old woman, who underwent brachial plexus transfer following an agricultural accident on the 9th of August, 2002. Herself a grazier and property manager, she suffered severe multi-trauma as her gloved hand was caught in the drive shaft of a commercial agricultural tractor-based pole digger. She became entangled in the drive shaft and was then rotated with considerable force. She experienced avulsion of her right arm and scapula, fractures to the right fifth to eighth ribs with pneumothorax, compound fractures of the left humerus, radius, and ulna, and a flail remaining arm from a complete brachial plexus injury on the left. Clinically then she had no right arm and a flail useless left arm.

Although without her right arm the subject did display good rhomboid, trapezius, and pectoralis function on the right. On the left the subject showed partial rhomboid function, with everything else below absent in terms of motor and sensory function to the left arm. Marked wasting of the left shoulder girdle was also apparent. Furthermore, there was early subluxation of the shoulder joint, Horner’s syndrome on the left as well as severe neuropathic pain relating to root avulsion in the left arm, alongside severe phantom pain on the right.

Eight days post-injury (17 August 2002), the patient underwent exploration of her right and left brachial plexuses. Pre-operative evaluation with MRI, EMG, and Nerve Conduction Studies had suggested that all the nerve roots and plexus on the right appeared relatively normal, whilst there was avulsion of the C7, C8, and T1 nerve roots on the left. At surgery it was confirmed that the nerve roots and plexus, as far as the cords where the amputation was on the right, were all completely normal. Unfortunately there was no arm for these normal nerves to innervate. On the left it was confirmed that the C7, C8, and T1 nerve roots were avulsed. Further, C6 was injured as far proximal as dissection was possible (i.e., at the foraminal level) and hence it was, in a clinical and surgical sense, functionally avulsed. In terms of the C5 nerve root, there was traumatic neuroma in continuity. It too was followed as far proximal as possible, divided millimeter at a time, however healthy fascicles were found to be irretrievable.

The patient recovered well from this initial 8 h procedure to identify the available nerves for a re-innervation procedure and after considering all options, the decision was made to proceed to re-innervate the left distal plexus by using the proximal plexus from the right to translocate it across the anterior neck and graft the proximal aspects of the right plexus to the distal components of the left.

A 15-h reconstructive procedure was undertaken 16 days post-injury (25 September 2002), where all plexus elements on the right and left were re-exposed. On the right, the cords were followed out to the amputation point and then trimmed. The medial and lateral pectoral nerves were mobilized to allow us to swing the plexus elements in front of the neck. The lateral, medial, and posterior cords were swung anterior to a point where the cut ends reached the midline of the neck. All the plexus elements were then exposed on the left. The full length of the left ulnar nerve was then harvested with its vascular pedicle. The following reconstruction was then performed:

- Right lateral cord to left lateral cord using 17 cm of vascularized ulnar nerve. A slip of this graft was sutured to the suprascapular nerve.
- Right radial nerve to left radial nerve using centimeter sural nerve grafts.
- Right axillary nerve to left axillary nerve using centimeter sural nerve grafts.
- Right medial cord to medial head of left median nerve using 17 cm vascularized ulnar nerve graft.

Following reconstruction the patient was reviewed regularly starting at 2- or 3-month intervals from November 2002 to November 2003. Subsequently she was reviewed in August 2004, December 2005, June 2006, and most recently in December 2012. The subject provided informed consent for all procedures and for the use of her clinical data for publication.

Functional MRI was conducted 3 years and 8 months after reconstruction, when the patient’s left arm examination was as follows: supraspinatus 3/5, deltoid 2/5, external rotation 0/5, biceps 4/5, brachioradialis 2/5, triceps 2/5, wrist extension 1–2/5, wrist flexion 4/5, finger flexion 4/5, thumb and finger extension 0/5, supination 3/5, and pronation 0/5. At latest follow up (December 2012) she had minimal sensory return but good motor return to flexor compartments. The patient reported major improvements in her phantom limb pain (PLP) but her left arm pain has remained unchanged since the accident.

Elbow flexion returned the quickest and is most certainly the most powerful movement to recover to date. At the time that functional MRI was performed, elbow flexion had become second-nature and could be performed without having to think about moving the non-existent right arm. Other movements still required a conscious effort to move the phantom right arm.

Functional imaging results show activity in the primary sensory and motor cortices after elbow flexion and finger-tapping. Plastic changes in the sensorimotor regions of the brain are studied so as to attain possible insights between instinctiveness of movement and level of functional changes elicited on functional MRI as possible forerunners for more permanent functional cortical rearrangement.

The patient was positioned supine in the MRI scanner and was able to view a high-resolution LCD screen located outside the bore of the scanner through a mirror located on the headcoil. Two identical blocked-designed fMRI experiments were completed separately; (a) finger tap and (b) elbow flexion. The experimental timings for both paradigms were the same and included 15 blocks, each of which were 30 s in duration. The total time for each experimental paradigm was 7 min and 30 s. The TR was set at 3 s thus 10 whole brain volumes were acquired during each block and 150 in total across the duration of each paradigm.

For the finger tap paradigm the patient was instructed that she would be see the words “REST” and “TAP” appear on the LCD screen. When “REST” was presented she was instructed to lie still and not move any part of her body and when the word “TAP” was presented she was instructed to tap her index finger and thumb together using an open and close pincer-like motion for the duration that the word tap was presented. The second paradigm was identical to the first but instead the word “FLEX” was used instead of “TAP” and accordingly the patient was instructed to elbow flex during this block. Both paradigms began and finished with a rest condition. The presentation of the visual stimuli were triggered by the scanner, at the beginning of each block and remained on the screen for the duration of the block.

Structural and functional imaging was performed on a 3T Philips Intera scanner (Philips, Best, Netherlands). Serial functional imaging consisting of 150 T2*-weighted blood-oxygen-level-dependent (BOLD) sensitive whole brain measurements were acquired using an EPI during the task performance. Each measurement consisted of 30 contiguous axial slices with the following scan parameters being applied: TR = 3000 ms, flip angle = 90, TE = 35 ms, field of view = 240 mm, slice thickness = 3.0 mm, matrix 96 × 96 giving an in-plane resolution of 2.5 mm × 2.5 mm, SENSE-reduction factor 2.2. Structural scanning consisted of a 3D T1-Magnetization Prepared Rapid Gradient Echo (MP-RAGE) sequence consisting of 182 contiguous slices of 1.0 mm thickness with an in-plane resolution of 0.97 mm × 0.97 mm.

All data analysis was conducted in the BrainVoyager QX software package (Brain Innovation, Maastricht, Netherlands) using existing analysis techniques described elsewhere (Berntsen et al., 2008). In brief, functional data were corrected for intra-session movement using a six-parameter model. Whole-volume global linear trends were removed, and the time series data were high-pass filtered (filter size = 90 s) to remove cardio-respiratory artifacts along with other cyclical influences of lower frequencies other than the paradigm presentation. The realigned functional time series were then co-registered to the skull-stripped 3D high-resolution T1-weighted structural images using an initial alignment based on image header position information followed by an intensity-based realignment algorithm for fine-tuning the co-registration, where the intensity-inverted first EPI-volume of each time series was least-square aligned to the 3D high-resolution T1-weighted image. Next, statistical analyses were conducted using contrasts according to the general linear model. Conditions were modeled according to a boxcar stimulus function convolved with a two-gamma hemodynamic response function. These contrasts were all calculated on mesh time courses, so that only voxels from the functional time series belonging to the gray matter of the cortical surface were included in calculations. Statistical parametric maps were corrected for multiple comparisons using the False Discovery Rate (FDR q: 0.05) and a cluster threshold of 50 mm^2^.

**Figure 1:**
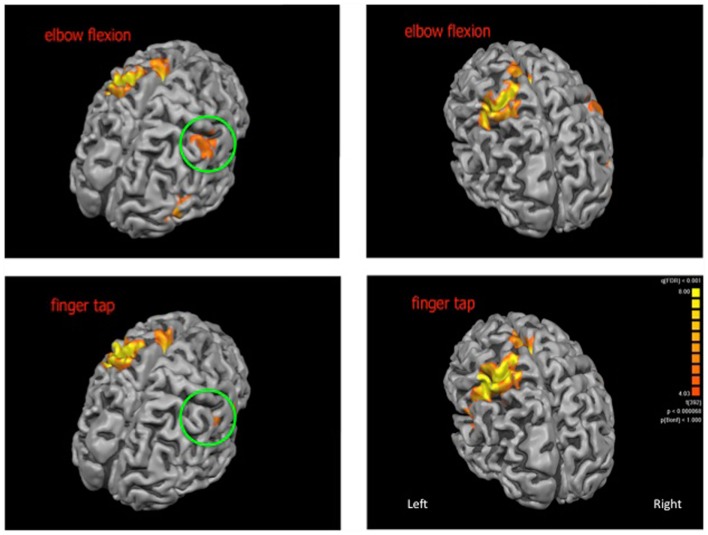
**Cortical activations (shown in orange) for elbow flexion (top panel) and finger tap (bottom panel)**. The patch of cortex exhibiting neuroplastic change is highlighted within the green circle. Cortical reconstructions are neurologically oriented.

For the elbow flexion task, significantly increased blood oxygenation level dependant signal was observed in the midline for the cerebellum and supplementary motor area as well as bilaterally in the thalamus, caudate nucleus, insula, and pre and postcentral gyri. For the finger tap task, significantly increased signal was observed midline for the cerebellum and supplementary motor area, bilaterally in the insula, pre and postcentral gyri and on the left side in the inferior frontal gyrus and thalamus (see Figure 1 for cortical activations and Table 1 for complete list of activated regions).

**Table 1:** **Regions of significant activations for elbow flexion and finger-tapping tasks**.

Region	Hemisphere	*x*	*y*	*z*	*Z*-score	# Voxels
**ELBOW FLEXION**						
Cerebellum	Midline	−2	−52	−39	5.23	8871
Thalamus	Right	21	−19	9	4.91	5309
	Left	−5	−16	8	3.85	4133
Caudate nucleus	Right	15	14	8	4.66	4551
	Left	−14	13	8	4.45	4736
Insula	Right	50	16	8	4.02	7508
	Left	−38	6	8	4.19	7119
Supplementary motor area	Midline	1	10	55	5.86	4693
Postcentral gyrus (sensory cortex)	Right	38	−23	55	5.02	3857
	Left	−42	−20	40	5.73	9487
Precentral gyrus (motor cortex)	Right	44	−6	40	5.69	981
	Left	−45	−8	42	5.42	9688
**FINGER TAP**						
Cerebellum	Midline	5	−53	−43	6.07	10052
Inferior frontal gyrus	Left	−36	35	18	3.87	3063
Thalamus	Left	−10	−18	5	4.73	4099
Insula	Right	50	9	11	4.81	6921
	Left	−38	−6	11	4.49	8768
Supplementary motor area	Midline	2	0	58	4.18	4557
Postcentral gyrus (sensory cortex)	Right	33	−14	46	4.88	1009
	Left	−44	−16	46	5.79	9737
Precentral gyrus (motor cortex)	Right	−45	−9	34	4.06	188
	Left	46	2	34	4.27	9516

*Localization of activated clusters was determined using Talairach Daemon (http://www.talairach.org/daemon.html)*.

## Background

Research has demonstrated that cortical plasticity, once thought only to exist in the early stages of life, does indeed continue on into adulthood (Flor, 2003). Brain plasticity is now acknowledged as a core principle of brain function and describes the ability of the central nervous system to adapt and modify its structural organization and function as an adaptive response to functional demand (Bach-y-Rita, 1990). As a consequence, brain function in adults remains adaptable and can change in response to peripheral events such as amputation, nerve block, spinal cord injury, or peripheral stimulation (Lotze et al., 1999). The functional organization of representational maps within sensorimotor regions, therefore is dynamic, reflecting the specific experiences of the organism with significant distribution between individuals (Changeux and Danchin, 1976; Plautz et al., 2000).

One form of functional adaptability within the brain has been outlined by Remple et al. (2001) who showed that behaviorally relevant stimulation and motor learning could lead to expansion of the topographical representations of trained areas within the motor cortex. These changes in mapping at the cortical level have been shown to reflect altered anatomy and physiology at the cellular level (Kleim et al., 1996). Synapse generation as well as dendritic arborization have both been found to be increased in rats following behavioral training on skilled motor tasks. Importantly however, cortical expansion occurs only when the movement and training is functionally relevant. To this end passive stimulation and simple movement does not elicit identifiable changes at the cortical level (Plautz et al., 2000; Remple et al., 2001).

Notable cortical rearrangement occurs following limb amputation or deafferentation. In such instances the brain’s motor and somatosensory homunculi are starved of input from the amputated limb and as a result, neighboring regions supplying intact structures expand, increasing their territory within the cortex. This process occurs rapidly, with studies showing that changes begin within just a few hours. This rapid time course implies that reorganization occurs not so much due to growing of new connections but rather via an alteration in the effectiveness of existing connections (Jacobs and Donoghue, 1991). Sensory deprivation to the cortex is an important factor in this form of plastic change. In a study conducted by Rossini et al. (1996) relating to partial hand sensory deprivation it was shown that excitability of cortical neurons adjacent to those deprived of their natural sensory feedback is unmodified or even enhanced compared with those neuron pools controlling muscles buried within sensory anesthesia. In the latter, excitability is significantly reduced. This drop in signal from the periphery may lead to reduction of inhibitory potentials to surrounding cortex eventually allowing for cortical plasticity (Hallett, 2001).

An interesting phenomenon relating to limb amputation and the repercussions of neuronal plasticity in the adult human brain is that of the phantom limb. Phantom limbs occur in 95–100% of individuals following leg or arm amputation and tend to resemble the somatosensory experience of the real limb prior to its amputation – giving the sensation that an amputated limb is still present. PLP on the other hand, occurs when this sensation manifests itself as pain in the form of cramping, burning, or other discomfort and is felt in approximately 50–80% of amputees (Melzack, 1990; Weeks and Tsao, 2010). PLP is thought, amongst other things, to be facilitated by the neuroplastic changes in cortical representation that occur subsequent to limb amputation. Indeed, previous studies have found that the degree of cortical rearrangement directly correlates with the severity of PLP (Flor et al., 1995, 2006; Lotze et al., 1999). Studies involving myoelectric prosthesis use, as a means of decreasing the degree of cortical rearrangement were undertaken and were for the most part effective at reducing PLP through provision of ongoing neuronal stimulation, visual feedback, and muscular training all of which are important in reducing, preventing, or reversing changes to cortical mapping (Ehrsson et al., 2004). The importance of visual feedback is further supported by the effectiveness of mirror studies in reducing PLP. In such studies patients, through the use of a mirror are able to create the illusion that a complete phantom limb has returned by reflecting the remaining limb (Ramachandran and Rogers-Ramachandran, 1996; Tsao et al., 2011). Sensory input, therefore, to the cortical networks that formally represented the now absent limb is important in reducing PLP.

Studies outlining reanimation of limbs are not uncommon and often involve injuries affecting cervical roots or brachial plexus. Cervical root avulsion, can be treated by nerve suture of the musculocutaneous and intercostal nerves. This technique has been found to be effective in restoration of function in the biceps brachii muscle and in so doing provides some degree of function to a previously immobile arm (Mano et al., 1995). Brachial plexus avulsions on the other hand are treated by nerve transfer of the biceps with fibers from the contralateral C7 root (Beaulieu et al., 2006). With regards the latter, Beaulieu et al. found that over time both ipsilateral and contralateral activations were elicited within the cortex as the brain sought to achieve conscious flexion of the ipsilateral arm (in which nerve transfer had been undertaken) via pathways that once exclusively brought about conscious extension of the contralateral arm. The former highlights the overcoming of the “synkinesis” phenomenon which occurs following the coaptation of the intercostal nerve and musculocutaneous nerves (Mano et al., 1995). It was found that the cortical motor area of the ipsilateral biceps had migrated to that of the intercostal muscles, with localization occurring once the ability to flex and control elbow flexion without simultaneous inspiration was achieved. These examples serve to highlight the plastic potential of the motor cortex in adapting to fulfill novel functional demands. Nonetheless, the supposed correlation between the degree of functional improvement and transposition activity on the motor cortex remains disputed (Sokki et al., 2012).

## Discussion

This study investigated functional activity in the sensorimotor cortex upon completion of elbow flexion and finger-tapping tasks in a formerly right-handed female subject with left-brain dominance that had undergone brachial plexus transfer. It was thought that the results of the neuroimaging procedures might highlight cortical changes of a neuroplastic nature in the brain and that the degree to which cortical activity was occurring might correlate with ease of movement. Cortical activation of the left hemisphere was maintained and was consistent between both tasks reflecting the incorporation of the right brachial plexus into the circuitry that now innervated the left limb. However, it was found that significant cortical activation of the contralateral hemisphere was occurring and was more pronounced during the elbow flexion task compared to finger-tapping. This was consistent with the patient’s account that the elbow flexion movement had in itself become second-nature. Indeed, at the time the patient reported no longer having to “think” that she was flexing her right elbow (now amputated) in order to achieve actual flexion in the left. In the case of the finger-tapping exercise, however, she described having to “concentrate” she was moving her right fingers in order to achieve finger tap in the re-innervated left hand. On this point, finger-tapping showed less activation in the contralateral hemisphere than elbow flexion. This may indicate that the degree to which cortical reorganization occurs is perhaps an indication of ease of movement. Nevertheless, this claim would require longitudinal follow up and a larger patient population for more certainty. In addition this finding may be confounded by the fact that increased cerebral blood flow within motor cortex is known to be correlated with increased force production (which may be expected in elbow flexion) (Remple et al., 2001) and furthermore elbow flexion may be a movement that is constitutively simpler to reproduce compared with finger tap.

Compared with conventional motor cortex localization within the brain, there was increased BOLD activation in the sensorimotor cortices following brachial plexus transfer which resulted in areas of cortex, contralateral to the re-innervated arm becoming active in addition to the ipsilateral cortical activation that was expected given the motor pathway to the left arm now included the right brachial plexus. This, potentially new, contralateral cortical activation could represent a degree of functional reorganization occurring in the right hemisphere as would be expected given that the left arm is being activated.

This plasticity is at odds with classical ideas relating to PLP which are based on the premise that the greater the degree of cortical rearrangement the worse the severity of the PLP (Flor et al., 1995, 2006; Lotze et al., 1999). This is incongruous with the present findings where the subject, who had experienced extensive PLP before surgery found these sensations were markedly reduced following transfer of the right brachial plexus to the still intact left arm. That which is worth noting in this case is that the cortical rearrangement that is occurring is useful, functional rearrangement. Indeed, the subject’s left arm through its re-innervation is receiving ongoing stimulation as well as behaviorally relevant muscular training while the brain receives the visual feedback of an intact limb. These are all critical factors which are important in mitigating PLP (Flor, 2003; Ehrsson et al., 2004). In the present study cortical activation was observed as a result of and in addition to the factors mentioned above; giving rise to remapping which is similar to that, which occurs in normal motor learning. It seems as though the continued stimulation of the functional brachial plexus with the visual feedback of the left arm is important in the diminution of the PLP experienced in this case as well as the motor function improvements. This gives rise to the question of self-attribution which represents another critical basis for reductions in PLP (Ramachandran and Rogers-Ramachandran, 1996; Tsao et al., 2011). The finding of mitigated PLP concomitant with the cortical reorganization noted might indicate that self-attribution may be occurring bilaterally, which indicates the brain is adapting to the new function assigned to the right brachial plexus. She has a normal image of her limb because she can see it, feel it, and move it, whilst the right brachial plexus is still actively employed and receiving sensory feedback. It may be that the body is adjusting, accepting its new parameters, and learning to deal with them. This is not out of the question given that Weeks and Tsao (2010) showed that incorporation of another person’s limb into one’s body image is possible as a method of relieving PLP.

These findings along with the reduction in phantom limb sensations noted post-transfer surgery, allow us to hypothesize as to the effects within the cortex itself as the brain adapts to the new function of the right brachial plexus and possible changes in self-attribution of the left arm. Another area of interest surrounds the existence of ipsilateral motor representations, supposedly present in the entire population, but with a high threshold for activation (Wasserman et al., 1994). The cortical rearrangement seen here may be due, at least in part, to these ipsilateral representations, believed to be mediated by interhemispheric projections via the corpus callosum (Kobayashi et al., 2003). Nonetheless, given that fMRI data from prior to the patient’s accident is unavailable, it is impossible to rule out the prospect that these areas of activation may have been present prior.

Finally it is important to note the heterogeneity between cases that exists in both brachial plexus and indeed neurological injury more broadly. An individual’s outcomes following such an injury (neurological or otherwise) can vary widely based on factors such as age, injury type, and severity, as well as latency between injury and repair (Mano et al., 1995; Furey et al., 2007). Higher IQ may also contribute to enhanced brain plasticity (Brans et al., 2010). Nonetheless the present study can serve as useful material for future clinical practice should patients with similar injuries present. Here the patient faced a situation of an amputated arm and one flail arm, and hence brachial plexus transfer seemed most viable. It should be noted that this procedure would not be indicated in all brachial plexus injury patients when function in the intact limb might show defects following attempts to re-innervate the recipient arm, which itself might not see any benefits (Krishnan et al., 2008). While the present study showed data taken some 3 years and 8 months post-surgery it might also prove fruitful to track the potential changes noted above over a longer time course to deduce the degree to which return of function is possible following such trauma and its subsequent treatment with brachial plexus transfer.

## Conflict of Interest Statement

The authors declare that the research was conducted in the absence of any commercial or financial relationships that could be construed as a potential conflict of interest.
